# Cost-Effective Wearable Indoor Localization and Motion Analysis via the Integration of UWB and IMU

**DOI:** 10.3390/s20020344

**Published:** 2020-01-07

**Authors:** Hui Zhang, Zonghua Zhang, Nan Gao, Yanjun Xiao, Zhaozong Meng, Zhen Li

**Affiliations:** 1 School of Mechanical Engineering, Hebei University of Technology, Tianjin 300130, China; hebut-zhanghui@outlook.com (H.Z.); zhzhang@hebut.edu.cn (Z.Z.); ngao@hebut.edu.cn (N.G.); xyj_hebut@163.com (Y.X.); 2 College of Automation Engineering, Nanjing University of Aeronautics and Astronautics, Nanjing 211106, China; zhenli@nuaa.edu.cn; 3 Nondestructive Detection and Monitoring Technology for High Speed Transportation Facilities, Key Laboratory of Ministry of Industry and Information Technology, Nanjing 211100, China

**Keywords:** indoor localization, motion analysis, wearable sensing devices, inertial measurement unit (IMU), ultra-wide band (UWB)

## Abstract

Wearable indoor localization can now find applications in a wide spectrum of fields, including the care of children and the elderly, sports motion analysis, rehabilitation medicine, robotics navigation, etc. Conventional inertial measurement unit (IMU)-based position estimation and radio signal indoor localization methods based on WiFi, Bluetooth, ultra-wide band (UWB), and radio frequency identification (RFID) all have their limitations regarding cost, accuracy, or usability, and a combination of the techniques has been considered a promising way to improve the accuracy. This investigation aims to provide a cost-effective wearable sensing solution with data fusion algorithms for indoor localization and real-time motion analysis. The main contributions of this investigation are: (1) the design of a wireless, battery-powered, and light-weight wearable sensing device integrating a low-cost UWB module-DWM1000 and micro-electromechanical system (MEMS) IMU-MPU9250 for synchronized measurement; (2) the implementation of a Mahony complementary filter for noise cancellation and attitude calculation, and quaternions for frame rotation to obtain the continuous attitude for displacement estimation; (3) the development of a data fusion model integrating the IMU and UWB data to enhance the measurement accuracy using Kalman-filter-based time-domain iterative compensations; and (4) evaluation of the developed sensor module by comparing it with UWB- and IMU-only solutions. The test results demonstrate that the average error of the integrated module reached 7.58 cm for an arbitrary walking path, which outperformed the IMU- and UWB-only localization solutions. The module could recognize lateral roll rotations during normal walking, which could be potentially used for abnormal gait recognition.

## 1. Introduction

With the rapid progress of Internet of Things (IoT) techniques, location is critical information for many fields and location-based services (LBSs) are widespread and prevalent in people’s daily lives [1,2,3]. Global Position System (GPS)-based localization and navigation and mobile base station positioning are the key building blocks for most location-aware services, such as driving navigation, drone navigation, animal tracking, and location-aware Internet searching [4]. The corresponding location information has allowed for an efficient and effective means to enhance people’s work efficiency, relieving efforts, or even provide intelligent service that was not possible in traditional ways. Based on the above facts, location-aware services have been playing an important role in a variety of fields, and it will continue to do so by integrating the ambient sensing and artificial intelligence (AI) techniques. Among the LBS solutions, indoor localization has also been a field that is continuously gaining research attention in recent years. Indoor localization techniques have attracted increasing attention in many applications, which are named indoor location-based services (ILBSs), including elderly care, robot navigation, sports motion analysis, rehabilitation medicine, smart buildings, etc. [5,6]. The commonly used techniques include wireless communication (WiFi, Bluetooth, ultra-wide band (UWB), and radio frequency identification (RFID)), optical positioning, inertial measurement, etc. For many application scenarios, the size, weight, power consumption, and non-line-of-sight (NLoS) of the sensing modules may be the critical concerns [7]. Normally, wireless communication navigation techniques, depending on a few pre-installed anchors or stations, have limited their fields of applications. In summary, a cost-effective, light-weight, compact sized, and energy-efficient wearable indoor localization module with the corresponding algorithms are a desirable solution for various indoor localization purposes.

Among the indoor localization techniques, the use of WiFi signal strength for fingerprinting-based methods has attracted much attention since WiFi is widely deployed as a wireless communication infrastructure. WiFi technology can achieve more complex large-scale positioning, but at the same time, has to deal with many other interferences [8]. The RFID positioning system consists of readers and tag devices, which is usually complex and the accuracy is not very high. Infrared positioning uses multiple infrared sensors placed in an indoor environment to measure the distance and angle of the signal source, thereby calculating the location of the moving node. This method can achieve a relatively higher accuracy for an empty indoor environment, but is susceptible to interference from indoor obstacles. Ultra-wide band positioning technology transmits and receives wireless data through narrow pulses, so it has the advantages of strong penetration, low power consumption, and high positioning accuracy. It is one of the indoor localization methods that are widely studied at present. The micro-electromechanical system (MEMS) inertial measurement unit (IMU)-based measurement with features such as a compact size, low power requirement, low cost, and being easy to use has gained extensive attention in recent years [9]. The IMUs normally estimate the attitude and position by measuring and fusing the information of three-axis acceleration, a three-axis gyroscope, and a three-axis magnetometer with noise cancellation algorithms, such as a Mahony complementary filter [10]; Kalman filter (KF) [11]; frame rotation calculation, such as Direction Cosine Matrix (DCM) or quaternion; and integral operations [12,13].

The inertial measurement for indoor localization suffers from cumulative errors in the integral operations, which cannot satisfy long-time localization applications. Most current investigations focus on the algorithms of noise cancellation and displacement calculations in advancing the accuracy of localization, which is limited by the performance of hardware [14]. Therefore, inertial measurement is usually integrated with other solutions for combined navigation in order to pursue a better performance. Some tentative investigations combining two or more techniques has been found, but most solutions suffer from bulky or expensive devices and low accuracy. Therefore, a cost-effective and light-weight wearable sensing device that is convenient for unobtrusive indoor localization and motion analysis is demanded at present. 

This investigation aimed to provide a cost-effective hardware solution with data fusion algorithms for wearable indoor localization and motion analysis. By comparing the alternative techniques, the UWB-IMU integrated solution was selected as the solution to take advantage of both UWB localization and IMU inertial measurement localization. Further investigations on identifying the underlying problems of the selected techniques and the integration of the two techniques were conducted with experiments and quantitative evaluations. The main contributions of this investigation are: (1) the design of a wireless, battery-powered, and light-weight sensing device integrating a low-cost UWB module-DWM1000 and a micro-electromechanical system (MEMS) IMU-MPU9250 for synchronized measurement; (2) the implementation of a Mahony complementary filter for noise cancellation and to calculate the attitude of moving objects, and quaternions for frame rotation to obtain the continuous attitude for displacement estimation; (3) the development of a data fusion model integrating the IMU and UWB data to enhance the measurement accuracy using Kalman-filter-based time-domain iterative compensations; and (4) experimental studies were carried out and quantitative evaluations were conducted to comprehensively evaluate the proposed solutions, and the results demonstrated the feasibility and the performance.

The structure of this paper is organized as follows: Section 2 gives a survey of the state-of-the-art indoor localization techniques and outlines the scope of this investigation. Section 3 presents the fundamentals of UWB localization, inertial measurement localization, and the proposed integrated solution. Section 4 illustrates the hardware and software implementations. Then, experimental studies are carried out and the results are discussed in Section 5. Finally, conclusions are drawn and future work is suggested in Section 6. 

## 2. Related Work

The huge demands of LBS devices in the future wearable IoT (WIoT) market has promoted investigations in the related areas, and a large number of investigations on indoor localization techniques, methods, and systems are reported in the literature [15,16]. New techniques and methods have been introduced to this field and combined methods have been proposed and practiced. As a result, the areas of applications are extensive. In this section, the novel technical solutions are summarized and discussed, the technical challenges are identified, and the scope of the investigation is outlined.

### 2.1. Indoor Localization Techniques: The State-of-the-Art

Many technical solutions are found to be used for indoor localization, which can be classified into five categories: (1) wireless communication, (2) optical/visual, (3) acoustic, (4) electromagnetic, and (5) inertial measurement [9,17]. Despite some technical solutions being competitive in terms of accuracy, such as ultrasound and optical/visual techniques, they still present problems in their adoption due to the cost of additional equipment and difficulties in the deployment and maintenance of dedicated infrastructure [18]. Therefore, the most commonly used techniques for indoor localization are: WiFi, UWB, RFID, and inertial measurement. A comparison of the most commonly used indoor localization techniques is given in Table 1.

(1) WiFi Fingerprinting Indoor Localization

**Figure 1:**
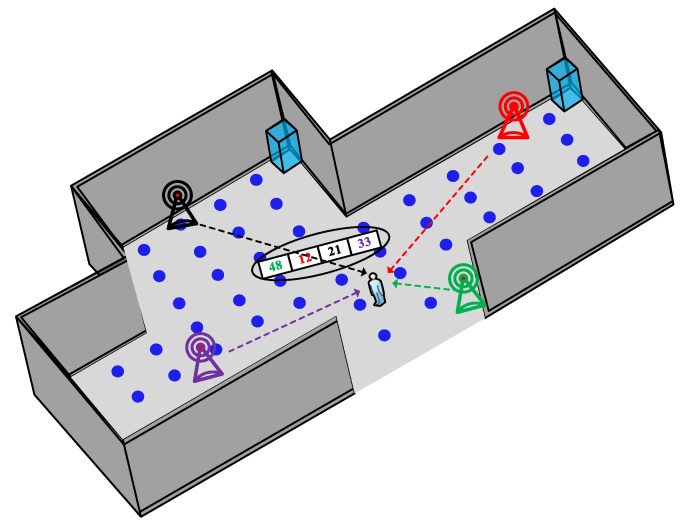
The fundamentals of WiFi fingerprinting indoor localization.

WiFi has been a widely used approach for indoor localization due to its advantages in NLoS, low cost, wide possession of WiFi enabled electronics among people, and convenient deployment by using existing WiFi infrastructure. WiFi fingerprinting, without knowing the exact access points (APs) locations, leads to a high feasibility in indoor deployment [1]. As shown in Figure 1, by comparing the fingerprints collected with the existing fingerprints in the database to estimate the locations of clients, the advantage of this method lies in its low cost and high reliability.

We have witnessed significant technical progress and plenty of applications in recent years. Zegeye et al. [23] implement WiFi received signal strength indicator (RSSI) fingerprinting and location estimation algorithms running on a server and a secure digital memory (SD) card of a mobile device. In order to manage the time-varying RSSI, Shu et al. [24] proposed gradient fingerprinting (GIFT) to leverage a more stable RSSI gradient, which first builds a gradient-based fingerprint map (Gmap) by comparing the absolute RSSI values at nearby positions, and then runs an online extended particle filter (EPF) to localize the user/device. GIFT has been shown to achieve an 80 percent accuracy of 5.6 m with dynamic WiFi signals. To handle the RSS fingerprint database, which is vulnerable to environmental dynamics, Zhou et al. [25] proposed WinIPS, a WiFi-based, non-intrusive indoor position system (IPS) that enables automatic online radio map construction and adaptation, which captures data packets transmitted in existing WiFi traffic and extracts the RSS and the media access control (MAC) addresses of both WiFi access points and mobile devices in a non-intrusive manner. Many other researchers have provided novel contributions to this field, including Gaussian process regression models [26], self-bootstrapping fine-grained passive indoor localization [19], frequency-diversity-based WiFi IPS [27], and digital navigation center IPS (DncIPS) [28], among others.

(2) RFID-Based Indoor Localization

As an object identification technique, RFID can be used for indoor localization due to the RSSI and phase of the radio signal, which can be collected by the RFID readers. Plenty of pioneer studies have been carried out and rapid technical progress has been made in recent years. One simple solution is to paste the RFID tags in fixed locations and let the moving object carry an RFID reader for localization. When the object moves, it receives the RFID tags, which can be used to lookup a pre-established tag-path position database and to calculate the corresponding location [29,30]. Another solution is phased array antennas localization, which applies multiple reading antennas to identify the phases of a tag in order to calculate its location. Wei et al. [31] applied the phase array antenna RFID localization to warehouse management and reached an accuracy of 3 m. Due to the reduction in the cost of RFID tags, many different applications have been found, such as smart storage item classification [21] and object localization on product shelves [32]. Further investigations about RFID localization are found in the literature, such as a multi-tag cooperative localization algorithm based on weighted multi-dimensional scaling [33], unwrapped phase-position model-based RFID localization [34], and a spatial-temporal phase profiling-based method for relative RFID tag localization [35]. The strength of RFID for indoor localization is NLoS, and the weakness is its low accuracy, high cost for the RFID reader, and complexity in the system setup.

(3) UWB-Based Indoor Localization

**Figure 2:**
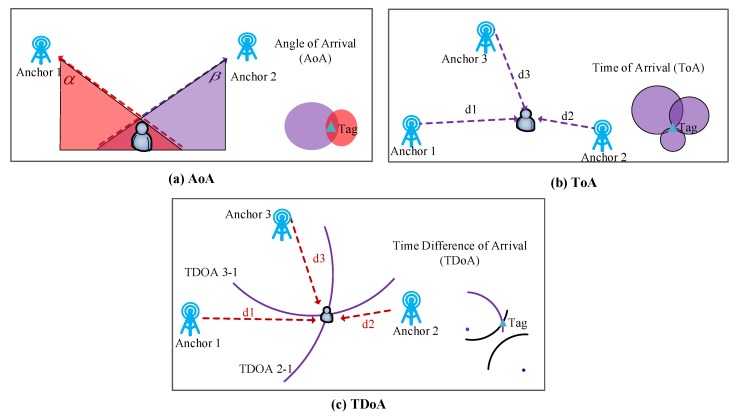
The fundamentals of acoustic and radio wave positioning.

UWB can be considered one of the most promising wireless technologies for localizing people or objects, with a high accuracy of about 10 cm. The methods used for UWB positioning are time of arrival (ToA), time difference of arrival (TDoA), and angle of arrival (AoA) [36,37], which are common for acoustic and radio wave positioning. The fundamentals are shown in Figure 2, where ToA localization is based on the ranging with a few nearby anchors, TDoA localization is based on comparing the time difference between the sensor node and each anchor, and AoA localization is based on the angles of the target node seen by reference nodes, which is achieved using antenna arrays [38]. Ridolfi et al. [39] presented investigations of the capabilities of UWB indoor localization systems for tracking athletes during their complex movements, which could be used to analyze the impact of on-body tag placement locations and human movement patterns on localization accuracy and communication reliability. Minne et al. [20] investigate the optimal position to mount the UWB hardware for in-depth analysis of the performance of athletes during training and competition, and obtained a median ranging error of 22 cm. To manage the performance degradation problem of UWB for NLoS localization, Yang [40] proposed a novel NLoS mitigation method based on a sparse pseudo-input Gaussian process (SPGP) with a low complexity. To improve the accuracy of the UWB-based indoor robot localization, Xu et al. [41] integrated an extended Kalman filter (EKF) and an extended unbiased finite impulse response (EFIR) filter, and the final estimate was obtained by fusing the outputs of both filters using probabilistic weights. According to the above investigations, UWB is considered a promising solution for indoor localization due to its accuracy. However, it is vulnerable to barrier blocking between moving nodes and anchors, which has limited its accuracy for NLoS occasions.

(4) Inertial Measurement-Based Indoor Localization

The MEMS IMU, due to its compact size and low power consumption, is attracting increasing attention for localization and motion analysis purposes. It estimates the location and tracks the motions through the in-built three-axis acceleration, three-axis gyroscope, and three-axis magnetometer. Plenty of related investigations have been reported in the literature. Yuan and Chen [42] presented a wearable human localization and motion tracking method using only three IMUs and obtained an accuracy of 0.1 m/s velocity tracking and 2% for localization. Kyritsis et al. [43] presented a convolutional neural network (CNN)-based algorithm for automatically detecting the in-meal food intake cycles using the inertial signals from an off-the-shelf smartwatch. Evangelos et al. [44] presented a gradient-based quaternion method to derive the direction of the arm segment, which can effectively detect and distinguish three elementary arm movements. Poulose et al. [22] presented a position-estimation algorithm that uses the combined features of data from an IMU sensor for position estimation, focusing on the pitch estimation, step detection, and heading estimation. The proposed pitch-based step detection algorithm achieves a 2.5% localization error, compared with acceleration-based step detection approaches that have a heading error of 4.72°. Zhang et al. [45] presented a novel indoor localization and monitoring system based on inertial sensors for emergency responders, which applies a zero velocity update (ZUPT) to reset the velocity within a still phase to deliver accurate position information. Zheng. et al. [46] presented a 3D indoor positioning system using a foot-mounted, low-cost MEMS IMU to obtain the position and attitude of a person in a 3D view. A ZUPT algorithm was developed to detect a standing still moment, and a Kalman filter was combined with ZUPT to eliminate non-linear errors in order to obtain accurate position information. From the above investigations, it is a common problem that the use of an IMU alone suffers from the position drift that grows exponentially due to the instability of sensor bias. The error cancellation techniques and algorithms are the key issues in the related studies. 

(5) Combined Indoor Localization Solutions

In addition to the above approaches, some investigations integrate two or more localization solutions to take advantage of the different techniques and optimize the performance. Inertial measurement is the most popular choice that is integrated with other solutions. Corrales et al. [47] used a Kalman filter algorithm to fuse the inertial motion system and the UWB positioning system, which can meet the requirements of tracking user actions and positions. However, the devices are bulky with wired connections, which is inconvenient for wearable applications. Wang et al. [48] integrated IMU and UWB devices and use particle filter algorithms for data fusion to achieve pedestrian positioning. Experimental results show that the algorithm can improve the positioning error to about 0.7 m, which is not ideal for many indoor localization applications. Sasiadek et al. [49] presented a sensor method based on a Kalman filter, which was applied to GPS/INS integration, and the universality of the method under different experimental conditions was discussed and verified. Murata et al. [50] presented a series of techniques that enhance a probabilistic localization algorithm, which utilizes mobile device inertial sensors and RSS from Bluetooth low energy (BLE) beacons. Experimental studies demonstrated the effectiveness of the proposed technologies to improve the localization accuracy from 3.0 m to 1.5 m. Jiang et al. [51] proposed a robot-assisted human indoor localization scheme utilizing acoustic ranging between a self-localized mobile robot and smartphones, and achieved an estimation accuracy of 0.43–1.12 m. Zhang et al. [52] integrated WiFi fingerprinting with IMU location estimation and achieved a localization error of 5.7 m over 5–10 min of indoor walking. There are also pioneer investigations that integrate UWB localization with IMU sensors for different purposes. Shi et al. [53] proposed an anchor self-localization algorithm for UWB and inertial measurement using an error-state Kalman filter (ESKF), which was utilized to fuse the UWB and inertial measurements. When UWB anchors are newly deployed, they are self-localized by freely moving an IMU-mounted UWB tag in the measurement space. Xu et al. [54] presented a human motion model based on geometric motion features. The model is applied to human lower limb motion sensing applications by combining the IMU sensor and UWB nodes to capture the Denavit-Hartenberg (D-H) parameters required for the proposed motion model. The test results showed that the positioning error and acceptable energy consumption were significantly lower than the conventional methods. Li et al. [55] presented an indoor positioning UWB/IMU fusion algorithm based on an adaptive robustness Complementary Kalman filter (CKF), which reduces the influence of an abnormal observation or abnormal system state on positioning results. Experimental results showed that this method had strong error recovery capability. Xu et al. [56] presented an optimization method based on the Chebyshev center, which is used to fuse the IMU and ToA information of the target positioning application. The proposed method can largely compensate against the error accumulation problem of IMU. The result was shown to have better performance compared with some existing methods. Zhong et al. [57] proposed a fusion positioning technology that combines UWB positioning technology and inertial navigation technology using a Kalman filter for LoS cases and found that the fusion positioning technology improves the positioning accuracy. 

### 2.2. Problem Statement

According to the commonly used indoor localization techniques discussed above, each technique may have its advantages and weaknesses for different indoor localization purposes. The WiFi fingerprinting is an economical and easily deployable choice that suffers from low accuracy due to time-varying RSSI. RFID is suitable for NLoS, while normally showing low accuracy and requiring a complex hardware system and inconvenient maintenance. Although UWB is competitive in accuracy, it suffers from NLoS interference and requiring multiple anchor deployment. Inertial measurement is usually lightweight and wearable, while the error accumulates with time, which may result in a very large error after long operation time. Some tentative investigations combining two or more techniques have been found, but most solutions suffer from bulky or expensive devices and low accuracy. Cost-effective and light-weight devices for unobtrusive wearable sensing is necessary for indoor localization. 

Due to the compact size and versatile functionality, IMU has become a popular choice for integration with other technical solutions for indoor localization. Although there have already been pioneer investigations reported in the literature, it still lacks a solution for indoor localization and motion tracking using the commonly used cost-effective electronics with detailed error cancellation and data fusion algorithms. This investigation aimed to provide a solution for indoor localization and human motion tracking by integrating UWB and IMU, which takes advantage UWB’s high localization accuracy and IMU’s NLoS localization and motion sensing. To validate the proposed solution, a UWB-IMU integrated compact and energy-efficient module was developed and the corresponding data fusion algorithms were designed and implemented. Finally, the whole system was verified with experimental studies. 

## 3. UWB-IMU Integrated Indoor Localization System

This section describes the principle and method of the positioning algorithms for both UWB and IMU solutions and the UWB-IMU integrated system regarding the hardware design and software algorithm. Then, the synchronized signal processing of the two kinds of sensor modules and the overall workflow of the system are presented in detail. 

### 3.1. Fundamentals of UWB and IMU Localization

(1) Fundamentals of UWB Positioning

**Figure 3:**
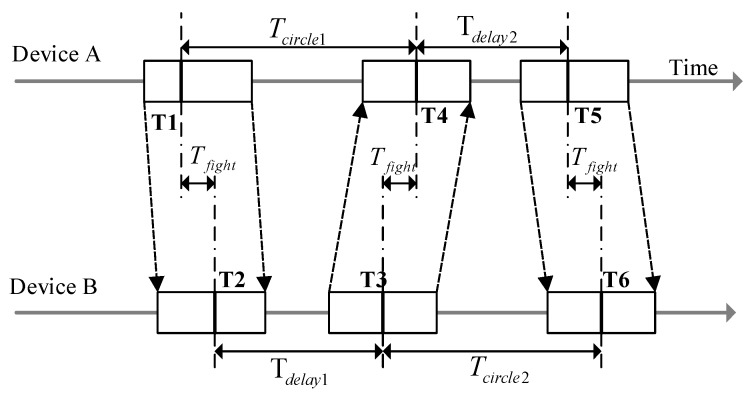
The fundamentals of the double-sided two-way-ranging (DS-TWR) method.

The UWB localization system realizes the positioning by measuring the distance between the tag and multiple anchors. The distance between the tag and anchor can be calculated by multiplying a signal’s time of flight (ToF) and the speed of light [58]. Therefore, accurately measuring the ToF of the signals becomes the key issue for UWB positioning. In this investigation, the double-sided two-way-ranging (DS-TWR) method [59], as shown in Figure 3, has been used for the localization.

As shown in Figure 3, device A sends a ranging message to device B. Then, device B receives the ranging message and waits for $T_{delay1}$ and sends a ranging message back to A. After receiving the ranging message from B, A waits for another delay $T_{delay2}$ and transmits the ranging message to B again. The timestamps of the messages that are sent and received by devices A and B in the above process are recorded. Finally, when device B receives the ranging message back form device A, the round-trip time can be obtained using Equation (1):

(1) $${\hat{T}}_{flight}\text{=}\frac{\left(T_{circle1}\;\times \;T_{circle2\;}-\;T_{delay1}\;\times \;T_{delay2}\right)}{\left(T_{circle1}\;+\;T_{circle2}\;+\;T_{delay1}\;+\;T_{delay2}\right)}$$

In Equation (1), the $T_{circle1}$ is time from device A transmitting the ranging message until device A receives a ranging message back from device B, and $T_{circle2}$ is the time from device B transmitting the ranging message until device B receives a ranging message back from device A. When the flight time between the anchor and the tag is obtained, the distance between these two can be obtained by multiplying the speed of the electromagnetic wave in the air. Then, the tag’s position can be estimated according to the positions of the anchors [60]. 

**Figure 4:**
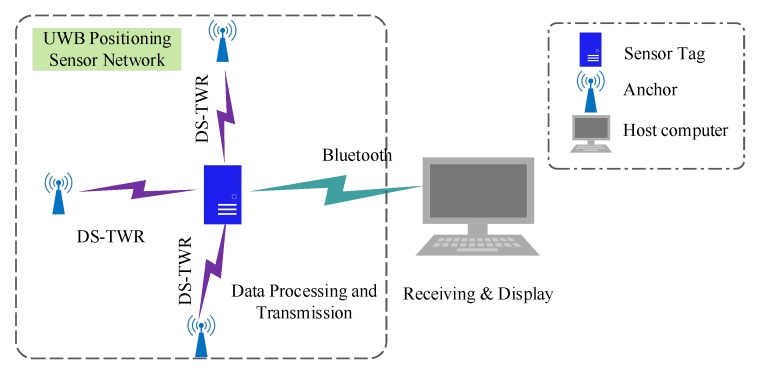
Schematic diagram of ultra-wide band (UWB) positioning system.

This UWB localization system contains at least three UWB anchors used for transmitting the ranging message from the tag, which are as shown in Figure 4.

(2) Fundamentals of IMU localization

**Figure 5:**
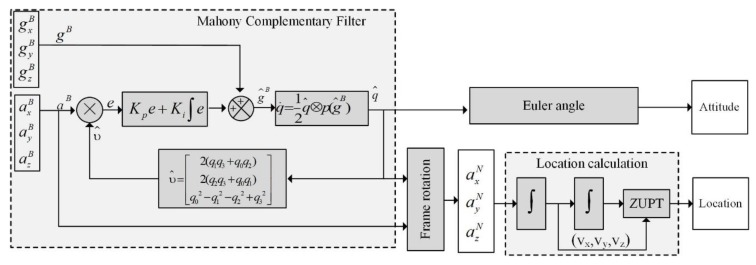
Schematic diagram of the inertial measurement units (IMU) attitude and position estimation. ZUPT: zero velocity update.

For IMU localization, we define two coordinate systems: the navigation coordinate N and the body coordinate B. The navigation coordinate N refers to the coordinate system referenced by the earth, and the body coordinate B is the coordinate system referenced by the IMU itself. Velocity and displacement are calculated by integrating acceleration in the navigation coordinate system. The acceleration in the navigation coordinate system is obtained by converting the quaternion with direction information obtained by the complementary filtering algorithm. The complementary filtering algorithm combines multiple sets of data and performs filter processing, and then outputs the final quaternion. The acceleration data has good static stability for attitude estimation, while it is relatively unreliable during dynamic motions. The gyroscope has better dynamic stability, but there might be a bias error, which results in a drift for long stationary times. Therefore, good dynamic performance with a small drift could be obtained by integrating the gyroscope and accelerometer outputs, which is shown in Figure 5.

In Figure 5, the three-axis acceleration measured by the accelerometer can be expressed with Equation (2), and the angular velocity obtained by the gyroscope is expressed with Equation (3):

(2) $$a^{B\;}=\;{\left[\begin{array}{ccc} a_{x}^{B} & a_{y}^{B} & a_{z}^{B} \end{array}\right]}^{T},$$

(3) $$\;g^{B}={\left[\begin{array}{ccc} g_{x}^{B} & g_{y}^{B} & g_{z}^{B} \end{array}\right]}^{T}.\;$$

The prediction $\hat{\upsilon }$ is the best estimate of the gravitational direction, which we take as being coincident with the Z-axis of body coordinate [61]:

(4) $$e\;=\;a^{B}\times \hat{\upsilon },$$

(5) $${\;\delta \;=\;K}_{p}{e\;+\;K}_{i}\int e,$$

(6) $$\;\dot{q}=\frac{1}{2}\hat{q}⊗p\left(g^{B}+\delta \right),\;$$

(7) $$\;a^{N}\;=\;\hat{q}a^{B}{\hat{q}}^{*}.\;$$

In the above equations, the normalized acceleration vector $a^{B}$ and the predicted direction $\hat{\upsilon }$ estimated with quaternion are used to obtain the compensation error *e* using their cross product. δ  is an innovation in the filter equation generated by a proportional-integral (PI) block. $\hat{q}\;$is the quaternion representation of the system attitude estimation and $\dot{q}$ is the rate of change of the quaternion, while p(·) is the pure quaternion operator (the real part of the quaternion is 0), meaning only rotation is considered. Converting the quaternion $\hat{q}$ to the angle output using the Euler angle, we can obtain the three angles representing the attitude.

In addition, calculating the displacement using the IMU requires a double-integration of the acceleration in the navigational coordinate system. The quaternion obtained in the previous step can help to convert the acceleration in the body system into the acceleration in the navigation coordinate system. In Equation (7), $a^{N}$ gives the accelerations in the N coordinate, $\hat{q}$ is the corrected gyroscope data expressed in quaternions, $a^{B}$ gives the accelerations in the B coordinate, and ${\hat{q}}^{*}$ is the conjugate of $\hat{q}$.

(8) $$v_{i}\;=\;v_{i-1}+\frac{1}{2}\;\times \;\left(a^{N}{}_{i-1}\;+a^{N}{}_{i}\right)\;\times \;\Delta t$$

(9) $$\;p_{i}\;=\;p_{i-1}\;+\frac{1}{2}\times \left(v_{i-1}+v_{i}\right)\;\times \;\Delta t\;$$

In this system, the IMU is mounted on the pedestrian’s foot, and the acceleration and angular velocity of the foot are measured in real time. One gait step is divided into foot touching and swing. When the foot touches the ground, the acceleration and velocity of the foot are considered to be zero. However, due to the noise of the IMU, the actual acceleration is not zero, which may introduce an error. We use the ZUPT algorithm [62] to separate the gait cycles and eliminate the error during the touchdown. The $a^{N}$ threshold is set for the acceleration in the N coordinate, and a small error of the acceleration within the threshold range can be interpreted as a constant speed motion (set to 0.025 m∙s^−2^ in this investigation). For acceleration values greater than the threshold range, the double-integration is used to calculate the position variation. The integration operations of acceleration for the position estimation is given in Equations (8) and (9), where $a^{N}$ stands for the acceleration, *v* for the velocity, *p* for the displacement, and Δt for the time interval. The sampling time Δt here needs to be small enough and remains constant.

### 3.2. The Designed System Based on IMU and UWB Modules

In this section, the change of position is obtained by accumulating the displacement in the X- and Y-axes. The following subsection presents the method for calculating the position with IMU data, and the data fusion model to integrate IMU and UWB data with a Kalman filter.

#### 3.2.1. IMU-Based Position Estimation with a Kalman Filter

Suppose the sampling interval is Δt, $p_{k-1}$ denotes the displacement in the X-axis direction at time (k − 1)Δt, and $Y_{IMU\_k-1}$ denotes the observation value of the IMU X-axis direction at time (k−1)Δt. An observation model is shown in Equation (10):

(10) $$Y_{IMU\_k-1}{\;=\;p}_{k-1}{\;+\;R}_{IMU},$$

where $R_{IMU}$ represents the displacement error of the IMU X-axis direction, and $R_{IMU}$ is obtained via statistical methods using a large number IMU observation samples. It is noted that the speed of the X-axis direction at time (k− 1)Δt is $v_{k-1}$, and the acceleration is $a_{x}^{N}$. The equations for motion with uniform acceleration are given as follows:

(11) $$p_{k}{\;=\;p}_{k-1}{\;+\;v}_{k-1\;}\Delta t\;+\;0.5(\Delta {t)}^{2}a_{x}^{N},$$

(12) $$\;v_{k}{\;=\;v}_{k-1}\;+\;\Delta ta_{x}^{N}.\;$$

At the time (k−1)Δt, the state variable $x_{k-1}$ is the displacement and speed in the X-axis direction:

(13) $$x_{k-1}\;=\;\left[\begin{array}{c} p_{k-1}\\ v_{k-1} \end{array}\right].$$

The equation of state can be obtained with:

(14) $$\left[\begin{array}{c} p_{k}\\ v_{k} \end{array}\right]\;=\;\left[\begin{array}{cc} 1 & \Delta t\\ 0 & 1 \end{array}\right]\left[\begin{array}{c} p_{k-1}\\ v_{k-1} \end{array}\right]\;+\;\left[\begin{array}{c} 0.5{\Delta t}^{2}\\ \Delta t \end{array}\right]a_{x}^{N}.$$

The observation equation of IMU is:

(15) $$Y_{IMU\_k-1}=\left[\begin{array}{cc} 1 & 0 \end{array}\right]\left[\begin{array}{c} p_{k-1}\\ v_{k-1} \end{array}\right]{+\;R}_{IMU}.$$

Therefore, the state space model can be given with Equations (16) and (17):

(16) $$x_{k}{\;=\;Ax}_{k-1}\;+\;Ba_{x}^{N},$$

(17) $$\;Y_{IMU\_k-1}{\;=\;Hx}_{k-1}{+\;R}_{IMU},$$

where $A\;=\left[\begin{array}{cc} 1 & \Delta t\\ 0 & 1 \end{array}\right],\;B\;=\left[\begin{array}{c} 0.5{\Delta t}^{2}\\ \Delta t \end{array}\right],\;\mathrm{and}\;H\;=\;\left[\begin{array}{cc} 1 & 0 \end{array}\right]$.

#### 3.2.2. UWB-IMU Data Fusion Model 

In this section, we integrate the position data obtained by the UWB and IMU. The position data of the IMU is obtained using integral calculation. The IMU calculation is relatively reliable for a short period, but its error accumulates over time. The UWB positioning may cause deviations due to problems, such as clock skew between the anchor and the tag. However, the measurement results do not drift over time. Therefore, we use the Kalman filter algorithm to integrate the two methods by using an iterative compensation of the two location results, and thereby achieve higher precision indoor positioning. 

**Figure 6:**
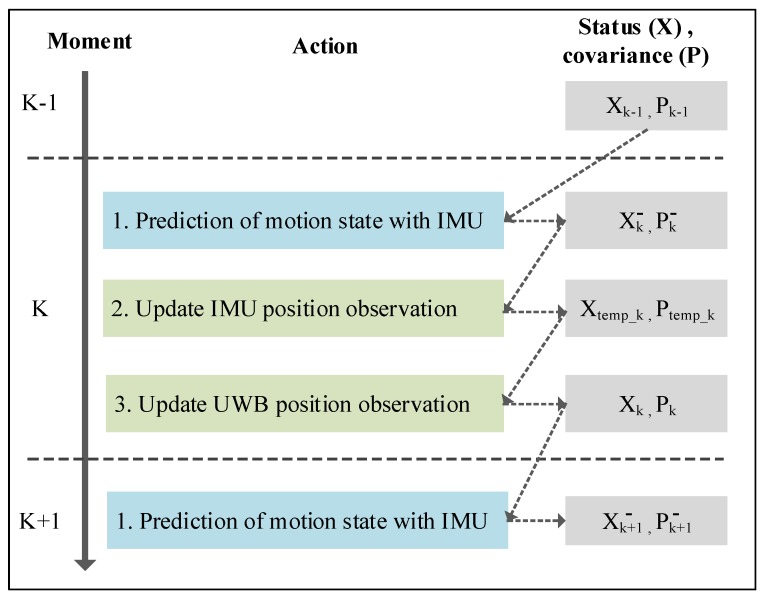
The data fusion process.

The data fusion model is shown in Figure 6. 

Based on the IMU observation data ($Y_{IMU\_1}\;$, $Y_{IMU\_2}\;$, …, $Y_{IMU\_k}$), the estimation of the pedestrian X-axis direction displacement $p_{k}$ can be obtained, and the calculation process is as follows.

(1) The current state is estimated based on the previous state using:

(18) $$x_{k}^{-}{\;=\;Ax}_{k-1}\;+\;Ba_{x}^{N},$$

where $x_{k-1}$ is the optimal estimation from the previous state and $x_{k}^{-}$ is the prediction of the current state.

(2) Calculation of the system covariance is done using:

(19) $$P_{k}^{-}{\;=\;Ap}_{k-1}A^{T}+Q,$$

where $p_{k-1}$ is the covariance matrix of the estimated value $x_{k-1}\;$and $P_{k}^{-}$ is the covariance of $x_{k}^{-}\;$. The *Q* matrix is the covariance of the motion model and it represents the error between the prediction model and the actual motion. The prediction model of this system is a non-uniformly accelerated motion. In a short interval, the interference caused by other influencing factors, including friction resistance and air resistance, is relatively small. The values of matrix *Q* are $\left[\begin{array}{cc} 0.1 & 0\\ 0 & 0.1 \end{array}\right]$.

(3) The Kalman gain transfer coefficient is updated using:

(20) $$K_{temp\_k}{\;=\;P}_{k}^{-}H^{T}{{(HP}_{k}^{-}H^{T}{+R}_{IMU})}^{-1}.$$

(4) The transfer status is updated using:

(21) $$x_{temp\_k}{\;=\;x}_{k}^{-}{+K}_{temp\_k}{(Y}_{IMU\_k-1}{-Hx}_{k}^{-}).$$

(5) The transfer state covariance matrix is updated using:

(22) $$P_{temp\_k}{\;=\;(I-K}_{temp\_k}{H)P}_{k}^{-}.$$

After updating the IMU observation equation, the state quantity $x_{temp\_k}$ and the covariance matrix $P_{temp\_k}$ of the IMU system at time *k* are obtained. The state estimation based on the IMU observation data is used as the prediction quantity in the UWB system, combined with the observations in the UWB system, to get the best position estimate. The state quantity $x_{temp\_k}$ and the covariance matrix $P_{temp\_k}$ of the IMU system are taken as the UWB system prediction state quantity $x_{k}^{-}$ and the system prediction covariance matrix $P_{k}^{-}$ make a status update, which are calculated as follows.

(6) The Kalman gain is updated using:

(23) $$K_{k}{\;=\;P}_{temp\_k}H^{T}{{(HP}_{temp\_k}H^{T}{+R}_{UWB})}^{-1},$$

where $R_{UWB}$ represents the UWB X-axis direction displacement error (observation noise), and $R_{UWB}$ is obtained via statistical methods using a large number of UWB observation test data.

(7) The status is updated using:

(24) $$x_{k}{\;=\;x}_{temp\_k}{\;+\;K}_{k}{(Y}_{UWB\_k-1}{-H\;x}_{temp\_k}),$$

where $Y_{UWB_{k}-1}$ represents the observed value of UWB localization at time *k* − 1.

(8) The state covariance matrix is updated using:

(25) $$P_{k}{\;=\;(I\;-\;K}_{k}{H)P}_{temp\_k}.$$

The state quantity $x_{k}$ and the covariance matrix $P_{k}$ of the system obtained after the UWB update are used as the fused output, and the two are used for the prediction process for the next iteration. 

### 3.3. Hardware Design and Data Synchronization

#### 3.3.1. Hardware Design

**Figure 7:**
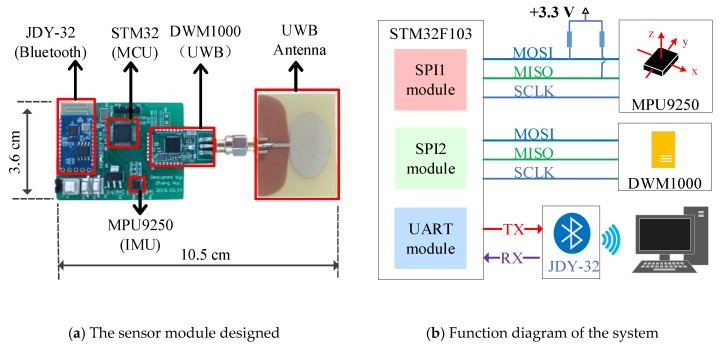
The UWB-IMU sensor module.

The integrated UWB and IMU positioning sensor module was designed with a STM32 (STM32F103C8T6, STMicroelectronics, Switzerland) micro control unit MCU as the central controller. The hardware circuit of the system included a STM32 MCU, a DWM1000 UWB module (DWM1000, Decawave, Ireland), a MPU9250 IMU (MPU9250, Invensense, USA), a JDY-32 Bluetooth module (JDY-32, Risym, China), and DC–DC module for the power supply. The designed sensor device and its functional diagrams are as shown in Figure 7a,b.

The DWM1000 is an ultra-wide band wireless module from DecaWave that complies with the IEEE802.15.4-2011 standard. In this system, the DWM1000 is responsible for marking the information transmission and reception time stamps and the communication between the base station tags and the sensor tag. The tag uses a polling method to communicate with the base station to complete the ranging function. The MPU9250 is a MEMS IMU module with a gyroscope, an accelerometer, and a magnetometer inside, which can measure the three-axis angular velocity, three-axis acceleration, and three-axis magnetic field. In this system, the acceleration and angular velocity of the sensor device are obtained using the MPU9250, and the corresponding displacement is used to complete the localization functions. Each time the DWM1000 and MPU9250 complete a set of data acquisition and calculations, the sensor device completes the calculation of the IMU position and the data fusion of the two sensing modules, and then sends the results to the host computer through Bluetooth. The host computer records the uploaded data and displays the graphics accordingly.

#### 3.3.2. Synchronized Processing for IMU and UWB Signals 

**Figure 8:**
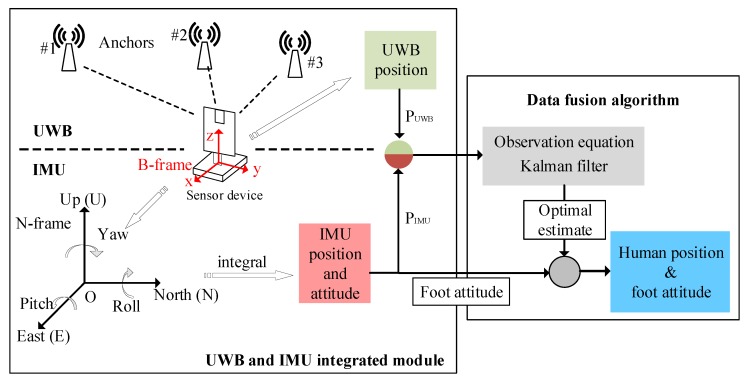
Synchronized processing for the UWB and IMU signals.

The synchronized data collection and processing of the two sensing modules is critical to the designed sensor device. When a motion is detected by the sensor device, the UWB obtains the displacement data via the DS-TWR method, and the angular velocity and acceleration from the IMU can be calculated to obtain the displacement and attitude. However, the displacement value calculated using the two sensors have certain deviations from the true value, so we compensate the two sets of data through a Kalman filter to obtain an optimal estimation of the displacement, which were presented in Section 3.2.2. The measured values of the two sets of sensors are used as inputs for the Kalman filter, which is used to find the positioning data closest to the true value. Finally, the optimal position estimate and attitude variation of the current time for the sensor device are obtained. The functional diagram is shown in Figure 8.

## 4. Experimental Verification and Results Analysis

In order to verify the feasibility and effectiveness of the proposed solutions in terms of both hardware and algorithm, we implemented the data processing algorithm with the designed sensor device. The data was collected through Bluetooth with a host PC recording and displaying the results using a MATLAB (R2016b, MathWorks, USA) graphical interface. Practical experiments of one-step walking, arbitrary path continuous walking, and abnormal gait detection were conducted for evaluating the proposed solution.

### 4.1. Measurement of One-Step Walking

**Figure 9:**
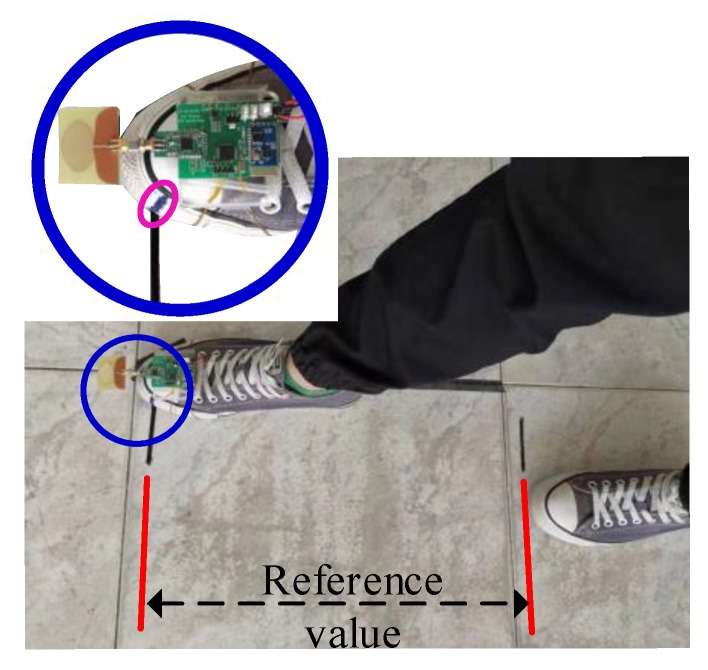
Displacement of one-step walking.

**Figure 10:**
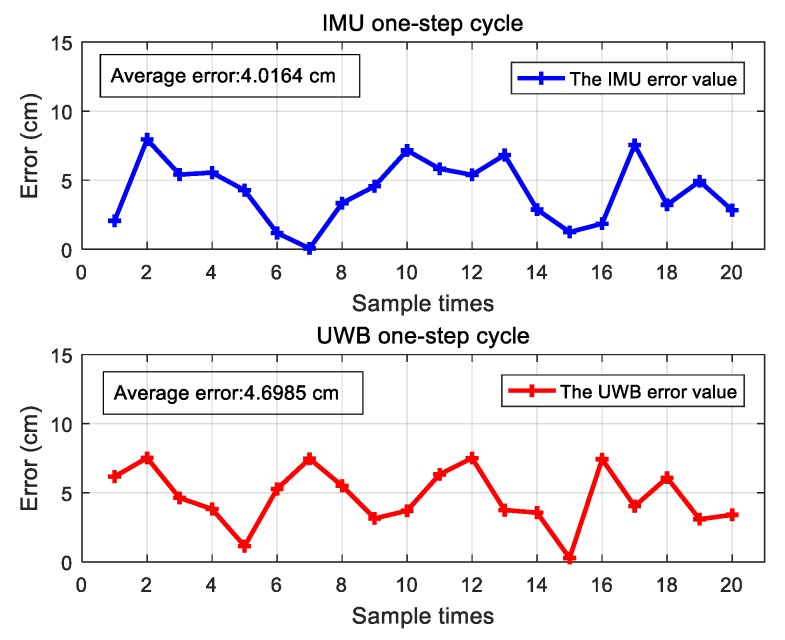
Errors of the 20 repeated walking steps.

**Figure 11:**
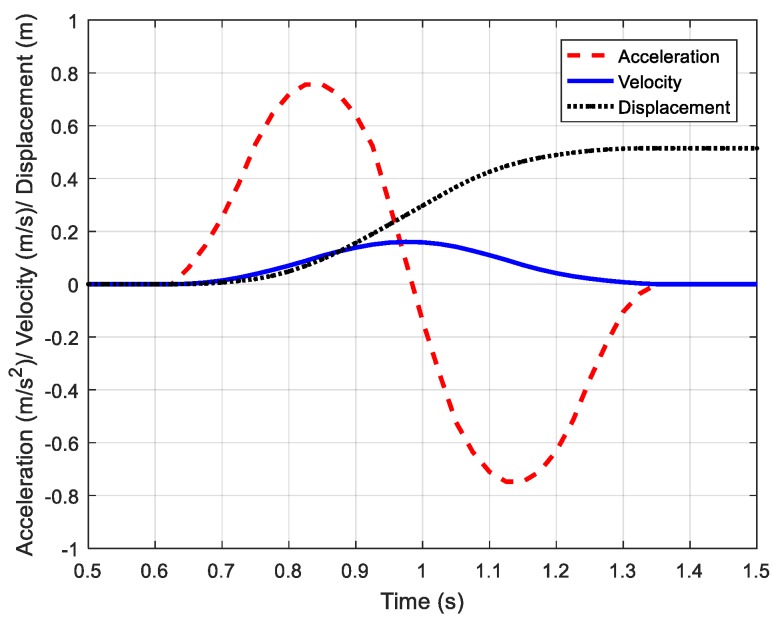
The acceleration, velocity, and displacement of one-step walking.

In this section, the positioning accuracy of the IMU module and the UWB module are evaluated. The sensor device was fixed on the surface of the foot and a one-step walking was performed 20 times to measure the step size. The experimental setup is shown in Figure 9. In order to obtain the walking distance, a marker point was made on the experimenter’s shoes, which is highlighted with the pink circle in Figure 9. The relative position of the marker point was used as a benchmark to obtain the displacement. The measurement results of the two modules for 20 repeated one-step walking events are given in Figure 10. The average errors of the IMU module and UWB module were 4.02 cm and 4.70 cm, respectively. Figure 11 shows the measured acceleration, velocity, and displacement during a one-step walking event with the IMU module.

### 4.2. Rectangular and Arbitrary Path Continuous Walking

#### 4.2.1. Experimental Setup

**Figure 12:**
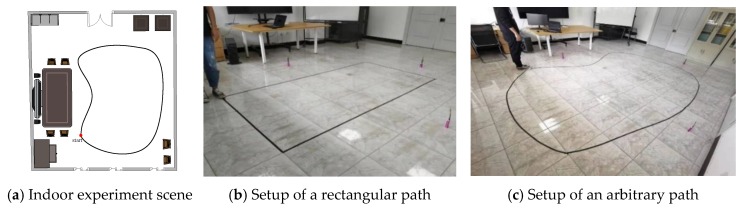
Indoor experimental scene and the experimental setup.

A meeting room sized 7.0 × 5.5 m including a few pieces of furniture was chosen as the indoor experimental scene for the further experimental studies, and the diagram of the room is shown in Figure 12a. In order to verify the localization accuracy of the fusion method with a Kalman filter by making use of the two sets of sensing data, experiments of walking along a rectangular path and an arbitrary path in an indoor environment were carried out. For the arbitrary path, it can be set to be much more complicated. However, the small corners in the complicated trajectory may be difficult to follow for the experimenter and large errors may be introduced. As shown in Figure 12, the black tape on the floor shows the paths for the experimenter to walk along. The initial position of the sensor device was defined the origin (0,0).

#### 4.2.2. Tests with a Rectangular Path

**Figure 13:**
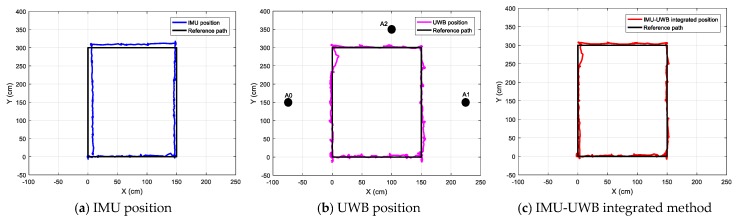
The measured locations for walking along a rectangular path.

By walking around the rectangular path once, the IMU-measured location, UWB-measured location, and fusion results are given in Figure 13. From the results, it is easy to find that:

(1) For the IMU measurement, there was an evident systemic error, as seen by the deviation between the black and blue lines in Figure 13a, which was hard to correct by the module itself. This was because the current location was calculated by integrating the variations of previous moments. However, the location was relatively stable and the error for one single step is not evident.
(2) For the UWB system, it was susceptible to NLoS occlusion of experimenter’s ankles, resulting in a bias error. As shown in Figure 13b, the positioning error caused by NLoS appeared multiple times in the UWB positioning results, and the error in the upper-left corner was significant. Although the localization accuracy was competitive for indoor applications, the results were not stable since the UWB radio was susceptible to ambient interferences.
(3) For the UWB-IMU integrated system, the advantages of the above two systems were combined. The UWB data was used to compensate for the inertial localization, and the inertial data was used to correct the UWB positioning data to make it more stable. The two sets of displacement data were iteratively compensated by the fusion algorithm, and as shown in Figure 13c, the final localization results were more accurate and stable.

#### 4.2.3. Tests with an Arbitrary Path

**Figure 14:**
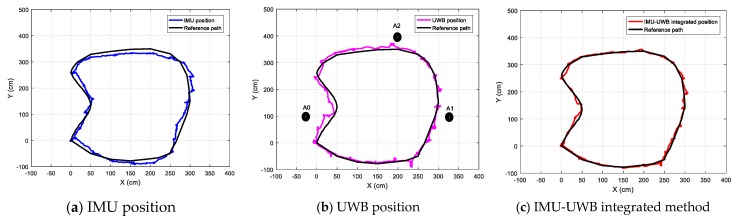
The measured locations for walking along an arbitrary path.

**Figure 15:**
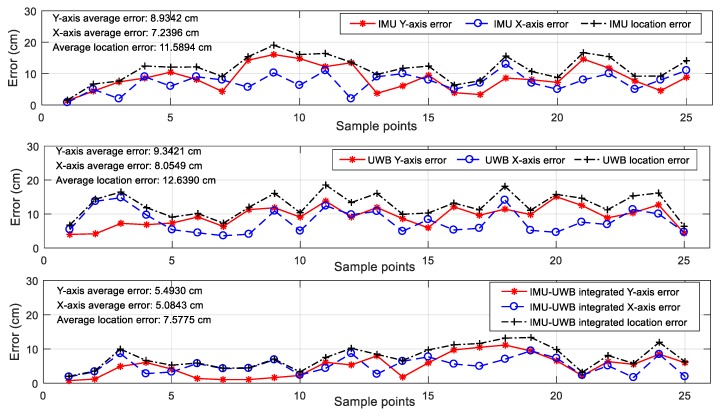
The measurement errors of the three methods.

For the arbitrary walking path, the location was obtained by marking the position of the foot, which is shown in Figure 9. The measured displacement was recorded by marking the foot positions for each step, and the measured values of the three methods by the sensor device were recorded and transmitted through Bluetooth to a host PC for evaluation and analysis. The final results are given in the figures below. Figure 14a–c show the measurement results of the three methods for the arbitrary path walking, and Figure 15 gives the X-axis, Y-axis, and overall position error between the measured value and the object positions for the three methods, respectively. From the test results, it is easy to find that: (1) the IMU localization suffered from a systemic error, and the low-cost IMU device could hardly obtain a high accuracy; (2) the UWB localization values were not stable due to sensitive radio wave signals; and (3) the UWB-IMU integrated method improved the localization performance in terms of both stability and accuracy. From Figure 15, the average position error ($\sqrt{x^{2}+y^{2}}$) of the IMU-UWB integrated method was 7.58 cm, which was significantly improved compared to 11.59 cm and 12.64 cm for the methods with IMU and UWB only.

### 4.3. Real-Time Attitude Measurement for Gait Analysis

**Figure 16:**
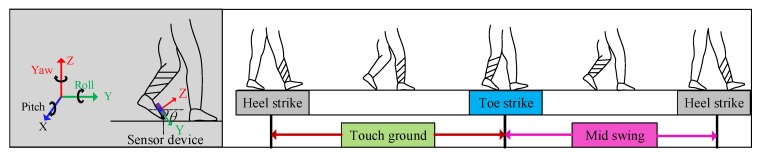
One gait cycle.

**Figure 17:**
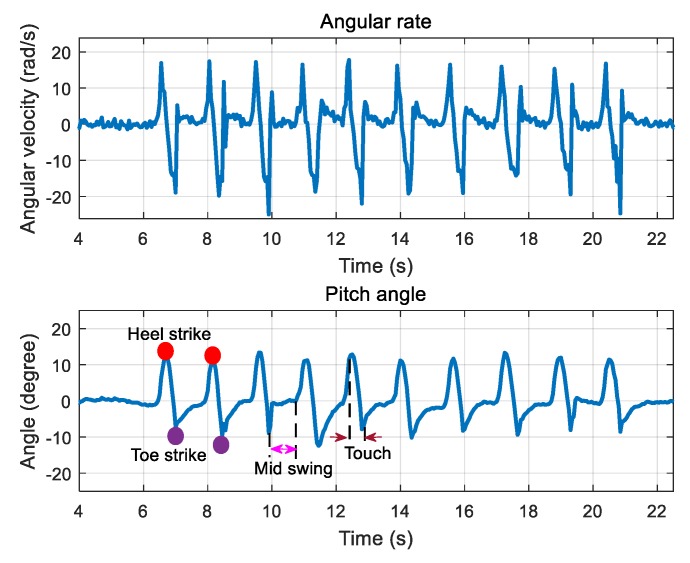
Measurement of foot attitude with the developed sensor device.

In addition to the indoor localization, the sensor device can also measure the attitude variation of the pedestrian’s foot during the walk. Since walking is a very common form of activity in human daily activities, it is a very important indicator for clinical rehabilitation. The gait shows how people walk and exhibit gait patterns that change periodically. A standard gait cycle can be divided into four phases, namely heel strikes on the ground, touching the ground, the toes touch the ground, and swinging, which are shown in Figure 16. In this system, we fixed the sensor device on the surface of foot to obtain the angle of the foot, namely the change of *θ* in Figure 16. As shown in Figure 17, the test results demonstrate that the change of the pitch angle in the normal gait period coincided with the actual changes in the posture of the foot surface.

**Figure 18:**
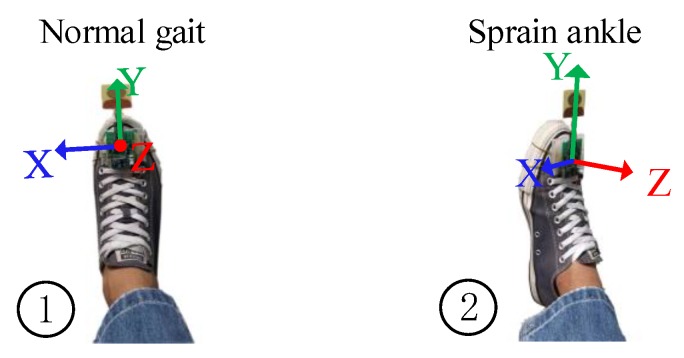
Normal and abnormal foot actions.

In order to prove the usability of measuring the angles for gait analysis, a test of simulated foot twisting during walking was performed, which is shown in Figure 18. When the sensor device was fixed to the surface of the foot and the foot twisting occurred, the abnormal actions (the foot twisting or falling) could be recognized by analyzing the roll angle. As shown in Figure 18, the roll angle fluctuated in a small range around zero degrees during normal walking and deviated from the normal threshold range once the abnormal foot action occurred.

**Figure 19:**
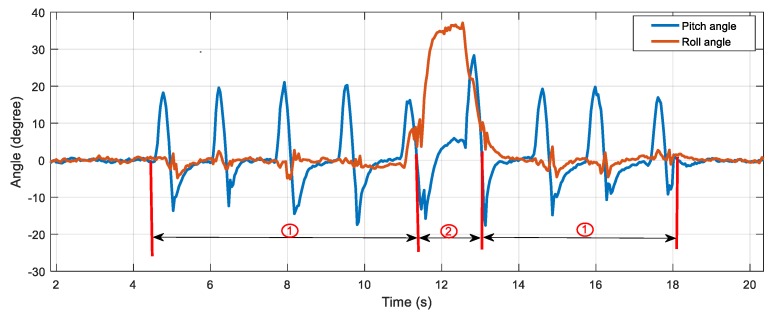
Measurement of abnormal foot attitude.

The results shown in Figure 19 demonstrate the potentiality of the developed sensor device for real-time gait analysis, which is of interest to many IoT applications. 

## 5. Discussion

By integrating the UWB and IMU with the proposed hardware and data fusion solutions, the UWB-IMU sensing module takes the advantages of the high-accuracy UWB localization and IMU localization signal stability, robust NLoS localization, and capability for motion tracking. Through the design and implementation in this investigation, the lessons learned are summarized as follows:

(1) For the IMU measurement, it was evident that the inertial measurement suffered from error accumulation although a ZUPT algorithm was implemented. The accumulated error finally turned to a system error in the inflection points, which decreased the accuracy of the final results. The possible solutions for correcting this system error might be to identify the inflection point and correct the inertial measurement result with a UWB measurement. This method may be able to effectively reduce the IMU accumulation error in the inflection point and promote the accuracy of localization results.
(2) The UWB measurement showed evident random errors, and there was also evident greater measurement errors when there was a NLoS occlusion between the sensor node and the UWB anchors. Being incapable of dealing with this error resulted in a lower localization accuracy. The promising solution to handle this error is to observe the gradient of the UWB location and IMU location and determine the emergence of barriers and correct the error by adjusting the trust weight of the UWB and IMU results.

Since indoor localization has attracted widespread interest in recent years, more accurate wearable modules and the corresponding algorithms are focal research topics. The solutions for the above technical issues may finally result in higher accuracy.

## 6. Conclusions and Future Work

### 6.1. Conclusions

This investigation proposed cost-effective hardware and data fusion solutions for the integration of UWB and IMU in a wearable module for human indoor localization and motion tracking. The Mahony filter and quaternions were employed for attitude estimation using the accelerometer and gyroscope, and the accelerations in the navigation coordinates were obtained to calculate the variations of location. UWB and IMU locations were integrated with a data fusion model based on a Kalman filter to obtain the final results. With these techniques, the designed module achieved the indoor localization and motion tracking goals. The UWB-IMU integrated solution takes the advantage of both the high-accuracy UWB localization and the IMU localization signal stability, robust NLoS localization, and motion tracking capability. Since the module is compact in size and is battery powered, it is a competitive alternative for many wearable indoor localization applications, such as elderly care, sport motion analysis, rehabilitation medicine, and robot navigation. 

### 6.2. Future Work

It was found that the UWB error caused by NLoS blocking by human body parts between the UWB anchor and sensor node had a critical influence on the measurement, and the accumulated error of the IMU measurement was another critical error. The future work will focus on seeking solutions for minimizing the errors, including:

(1) A smart algorithm to automatically identify the NLoS blocking errors and adjust the weight of trust between the UWB results and IMU results to improve the localization accuracy.
(2) The determination of the key turning corners in the motion where the IMU results may introduce errors and use the UWB location to correct the accumulated error of IMU localization results.
(3) Comprehensive evaluation of the designed IMU-UWB system by comparing the estimation with more reliable references using methods such as the Cramer–Rao low bound (CRLB).
(4) Evaluation of the influence caused by indoor environment including human body and furniture, such as the error caused by the random walking pedestrians and their walking speed.

In addition, to extend the UWB-IMU measurement from indoor localization to building-scale localization and navigation for product and human tracking is a promising field. Undoubtedly, there has been a great need for indoor localization for human tracking and motion analysis. A smart wearable sensor device that is more accurate, reliable, and easy-to-use for indoor localization is of interest to many novel IoT applications in the future rich-sensing smart world.

## Figures and Tables

**Table 1:** The commonly used indoor localization techniques.

Method	Technology	Accuracy	Pros	Cons
WiFi [19]	* RSSI fingerprinting	1–2 m	Low cost, simple system	Data base required for fingerprinting, low accuracy
UWB [20]	^†^ ToA/TDoA/AoA	0.1–1 m	High accuracy, simple system	Short range problems in NLoS
RFID [21]	RSSI	Connectivity range	Low power, low cost	Low accuracy, one tag per location, complex system
Inertial measurement [22]	Acceleration, angular velocity, magnetometer	1–5% of the traveling distance	Compact size, low cost, NLoS ^#^	Position/orientation drift, magnetic disturbance, accumulated error in calculation

* RSSI: Received signal strength indicator; ^†^ ToA/TDoA/AoA: Time or arrival/time difference of arrival/angle of arrival; ^#^ Non-line-of-sight.
